# An ethnobotany survey of wild plants used by the Tibetan people of the Yadong River Valley, Tibet, China

**DOI:** 10.1186/s13002-022-00518-8

**Published:** 2022-03-31

**Authors:** Chang-An Guo, Xiao-Yong Ding, Yi-Won Addi, Yu Zhang, Xiao-Qian Zhang, Hui-Fu Zhuang, Yu-Hua Wang

**Affiliations:** 1 Yunnan Key Laboratory for Wild Plant Resources, Kunming Institute of Botany, Chinese Academy of Sciences, 132# Lanhei Road, Yunnan, 650201 Kunming China; 2 University of Chinese Academy of Sciences, Beijing, China

**Keywords:** Eastern Himalayas, Biodiversity hotspots, Tibetan, Traditional knowledge, Yadong county, Culture exchange

## Abstract

**Introduction:**

Plant resources gathered from the wild are important sources of livelihood needs, especially for low-income populations living in remote areas, who rely on these plants for food, fuelwood, medicine and building materials. Yadong County is a valley at the border between the China, India and Bhutan in southern Tibet. Yadong is rich in biodiversity and culture, but ethnobotanical knowledge has not been systematically studied. This study aimed to document the ethnobotanical knowledge of Tibetans in Yadong County.

**Methods:**

Ethnobotanical data were documented through free listings, key informant interviews, and semi-structured interviews during fieldwork. The culture importance index (CI) and informant consensus factor index (FIC) were used as the quantitative indices.

**Results:**

In total, 163 informants (46 women and 117 men). A total of 3,031 use reports and 121 plant species belonging to 52 families and 91 genera were included. These use reports were then classified into 20 categories belonging to 9 major categories. The utilisation category that containing the most plant species was food, followed by economic, medicine, animal feed, social uses, other uses, environmental uses, materials and fuels. Among the economic plants, 32 medicinal plants are traditionally used in the local region. Plants with high CI included *Fritillaria cirrhosa*, *Neopicrorhiza scrophulariiflora*, *Betula utilis*, *Rheum nobile*, and *Urtica hyperborean*.

**Conclusion:**

This research demonstrates the diversity of the types and functions of Yadong Tibetan traditional plant knowledge. Knowledge of edible and medicinal plants in this area is prominent, reflecting the ability to cope with the lack of fruits and vegetables and basic family medical care. There were exchanges between the traditional plant culture in the study area and its surroundings. With socioeconomic development, the commercial value of medicinal plants has increased, and locals are also seeking ways to adoptsustainable development to cope with the excessive consumption of plant resources.

## Introduction

Wild plant resources are important sources of food, fuelwood, medicine, forage and building materials, especially for the poor, living in remote areas [1]. However, the traditional understanding of wild plants is rapidly lost due to the development of socio-economic development [2]. Moreover, traditional knowledge depends on specific locations and information passed down through generations [3]. Thus, documentation and evaluation of traditional ethnobotanical knowledge are urgently needed [4].

Yadong County is located on the edge of biodiversity hotspots in the eastern Himalayas, India, and Myanmar [5]. The Yadong River runs through Yadong County and presents a valley topography; Yadong County is also called Yadong River Valley and is an important border area in southern Tibet bordering Bhutan and Sikkim to the east, west, and south and has 41 mountain passes connecting Yadong to Bhutan and Sikkim. Because of its proximity to the sea and its superior border trade environment, it has become the largest port for border trade in Tibet. In addition, trade between Nepal, India and China is active [6].

Tibetans live mainly on the Qinghai-Tibet Plateau. They have a long history and rich traditional cultural knowledge in many aspects such as food, medicine, religion, architecture, and handicrafts [7]. Although Tibetans are classified in China as a minority, they consist of several Tibetan languages, dialects and ways of life, with six main groups/dialects, three of which occur in China and are classified as Ü-Tsang, Kham and Amdo [8]. The language used by Tibetans in Yadong belongs to the Ü-Tsang dialect in the Tibetan branch of the Tibeto-Burman language group [9]. The main sources of income for the local population are livestock rearing and gathering herbs. Nomads seasonally migrate to high altitudes during the summer with their yaks and return to permanent settlements before the onset of the winter. Herbal gatherers usually harvest during spring and summer when plants grow. Seasonal labour across altitudes is part of their lives.

The main Tibetan settlements abroad are in India, Nepal, and Bhutan, most of which are in the southern foothills of the Himalayas. Foreign ethnobotanical research is mainly concentrated in these countries and regions, and related research involves the utilisationof edible plants, medicinal plants, handicrafts, dyeing, feed, and fibres. Tibetans live in vast area of China, including Tibet, Qinghai, western Sichuan, and northwestern Yunnan. However, ethnobotany research on Tibetans in China is mainly concentrated in some provinces and cities in the eastern Tibetan region. In most Tibetan areas, ethnobotanical research is still lacking.

Tibetans have a rich and unique knowledge of the local environment, such as climate, soil, wildlife, vegetation, and plant utilisation[10]. This traditional knowledge stems from the interrelationships among humans, plants, animals, natural phenomena, and religious beliefs. Predecessors have conducted surveys in Tibet and other areas where Tibetans live and have found that some wild plants have provided many products and services for locals. Traditional Tibetan medicine has been the main disease treatment in many remote areas, relying on a large number of wild plants as well as traditional treatment methods [11–30], and some of these medicinal plants are also collected for trade [26, 28, 31–34]. Various wild edible plants have been collected for food [23, 24, 32, 33, 35–41]. Tibetans also place flowers collected from the wild on the altars of houses and temples or collect Tibetan incense plants to worship their gods [25, 42]. In addition, there is a wealth of knowledge on forage, house building, tools, fuels, dyes, and seasoning [11, 25, 43].

However, to date, traditional knowledge of plants used by Tibetans in Yadong has not been explored. Therefore, this research aimed to document the ethnobotanical knowledge of Tibetans in Yadong County.

## Method

### Study area

**Fig. 1:**
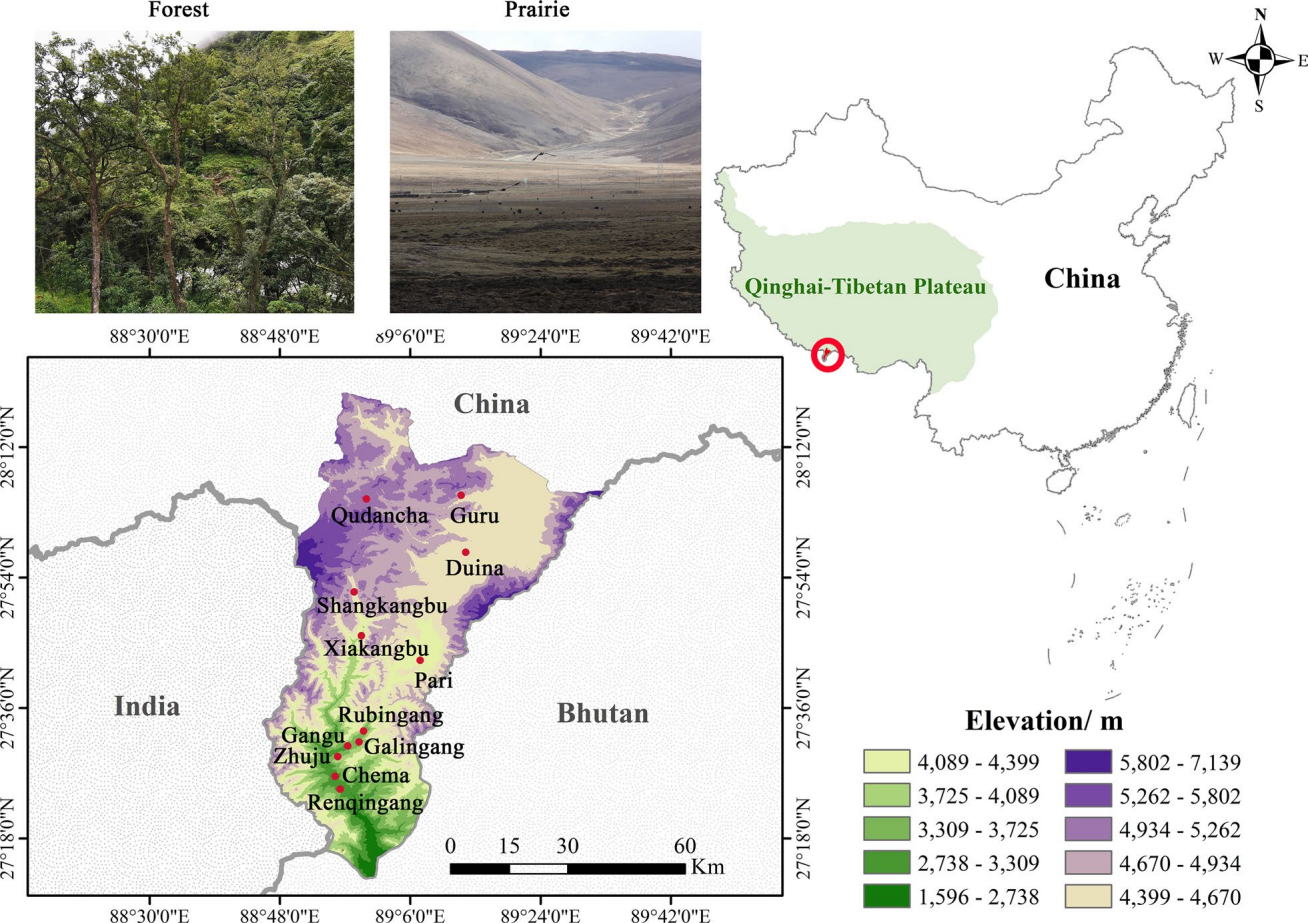
Map of the study area

Yadong County, which belongs to Shigatse City, Tibet, is located in the southern Himalayan mountains and borders India and Bhutan (Fig. 1). The altitude ranges from 1,600 to 7,300 m, and the annual average temperature is 0 °C, with the average temperatures of the coldest and hottest months of -5 °C and 10℃, respectively [44]. The terrain is high in the north and low in the south. The northern part of Yadong is an important plateau pasture in Tibet, and is mainly composed of alpine ecosystems. The southern part of Yadong has a large area of virgin forest, which has a mild climate and abundant water resources, and is considered a green treasure house in the Himalayas. The total population of Yadong County is 13,992, among which Tibetans account for 98%, Han 1.5% and other minorities 0.5% [44]. Economically, the Yadong District can be defined as a rural area based on agricultural and livestock activities. *Hordeum vulgare* var. *coeleste* is the predominant crop, and *Bos grunniens* is the main livestock [45–47].

### Data collection

In August 2020 and May 2021, our ethnobotany fieldwork was conducted on 12 villages of 7 townships in Yadong (Fig. 1). First, field study permission was obtained from the local community committee and government authority. We explained our purpose to local governments and requested assistance from them. All our fieldwork was conducted with informed consent.

The snowball sampling method was used to select the key informants, such as veterinarians and herdsmen. Other informants were selected by the randomized household interview method. In total, traditional knowledge was collected from 163 informants. Ethnobotanical knowledge was collected by semistructured face to face interviews. Because many Tibetans in the study area cannot speak Mandarin fluently, the field work was performed with the assistance of local guides who were employed with the help of local community leaders. All interviews were conducted in the Tibetan language, which was translated into Mandarin by local guides. All field studies were conducted with the consent of informants. According to the commonly used 5 w + 1 h (What, Where, When, Who, Why, How) principle in ethnobotany, this study designed the following questions for semistructured interviews:

1. Would you mind listing some wild plants you have used?
2. How to use this plant?
3. Which plant parts were used, roots, stems, leaves or other parts?
4. Why do you use this species?
5. What time do you collect this plant?

The questions were designed to collect data on the (i) vernacular name of the plants, (ii) category of use, (iii) parts used, (iv) methods for preparation and administration, (v) characteristics of the plant material (dried or fresh) and (vi) collection time.

The specimens were collected from the field of survey with the help of the key informants and all materials are labelled with numbers and names. Photographs of each plant were taken. All specimens were kept in the herbarium of Kunming Institute of Botany (KUN). The Flora of China was used to help identify the plants [48] and The Plant List was used to ensure the Latin name of the plants [49].

### Data analysis

We adopted the Use report (UR), cultural important index (CI) and informant consensus factor index (FIC) as ethnobotanical indices. All information about the use of local plants was organized into a “use report” list consisting of three parts: informant, used plant and used category [50, 51].

The cultural important index (CI) [52] was the sum of the proportion of informants that mentioned each of the use categories for a given species. This index is used to quantitatively evaluate the importance of a certain plant to Yadong Tibetans from the perspective of comprehensive value. In other words, CI represents the diversity of plant uses and the degree of recognition of information sources for each use category. The calculation formula is as follows:

$$CI={\sum }_{U=u1}^{uNC}{\sum }_{i=i1}^{iN}\frac{URui}{N}$$

NC was the total number of use categories and N was the total number of informants. CI ranges between 0 and the number of all utilization categories. A higher CI value indicated the multiple uses of a species and a higher degree of recognition.

The informant consensus factor index (FIC) was developed by Robert T. Trotter [53]. FIC was used to evaluate the degree of consensus among the population about how to treat a particular disease. The calculation formula is as follows:

$$FIC=\frac{Nur-Nt}{Nur-1}$$

where Nur is the number of use reports from the informants for a particular disease and Nt is the total number of plant species used to treat the disease. The FIC values range between 0 and 1. A higher FIC means that different herbalists have a higher consensus on the plant species used to treat certain diseases.

## Results

### Distribution of knowledge among informants

**Fig. 2:**
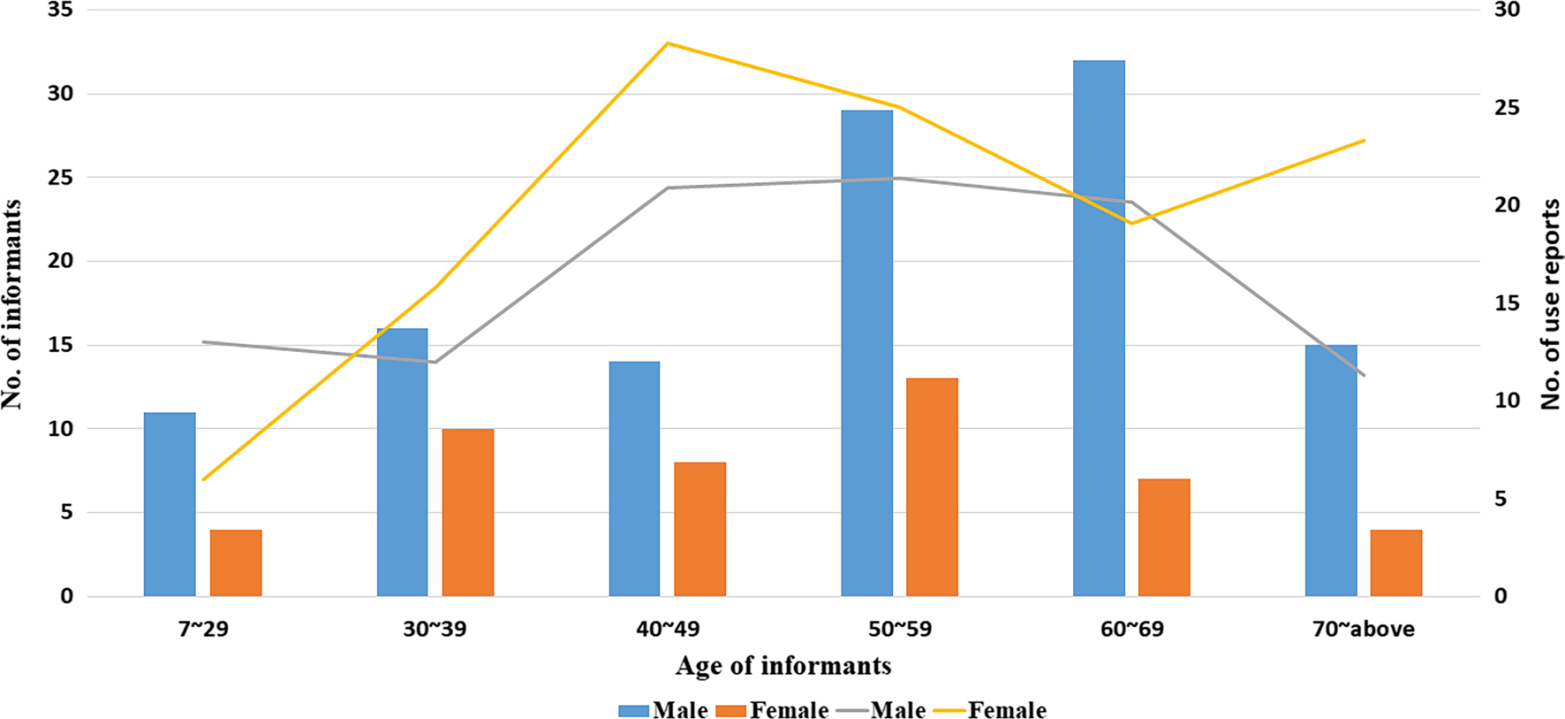
Distribution of knowledge among informants: The line represents the average number of URs provided by local people in each age group

This study documented 3,028 use reports (UR) from 163 informants. Among the informants, 46 were women and 117 were men (Table 1). The selection of our information reporter was random but resulted in more men and fewer women. This reason may be because the right to speak is mainly in the hands of men, who are primarily responsible for external affairs and livelihoods in most families, while women are mainly responsible for household affairs [54]. The informants were aged from 7 to 81 years, the average age was 52 years, while that of men was 53 years, and 49 years for women. Middle-aged individuals (40–59 years) provided the most use reports on plant use, whereas young individuals under 30 years old and the elderly over 70 years old provided fewer Urs than did middle-aged people (Fig. 2).

**Table 1:** Characteristics of informants

Characteristics	Number	Percentage
*Communities*		
Qudancha	13	8.0
Guru	12	7.4
Duina	13	8.0
Pari	17	10.4
Shangkangbu	18	11.0
Xiakangbu	17	10.4
Gangu	17	10.4
Rubingang	10	6.1
Galingang	9	5.5
Zhuju	8	4.9
Chema	10	6.1
Renqingang	19	11.7
*Gender*		
Female	46	28.2
Male	117	71.8
*Age*		
Below 29	15	9.2
30–39	26	16.0
40–49	22	13.5
50–59	42	25.8
60–69	39	23.9
Above 70	19	11.7

### Taxonomic diversity of wild plants used by locals

In total 121 plant species belonging to 52 families and 91 genera were identified in the study area. The most cited family was Compositae (16 species), followed by Rosaceae (9), Polygonaceae (7), Ericaceae (6), Apiaceae (5) and Lamiaceae (5) (Table 2). Among the plants, 89 were herbaceous, 20 were shrubs, 10 were trees, and 2 were vines (Table 2).

**Table 2:**
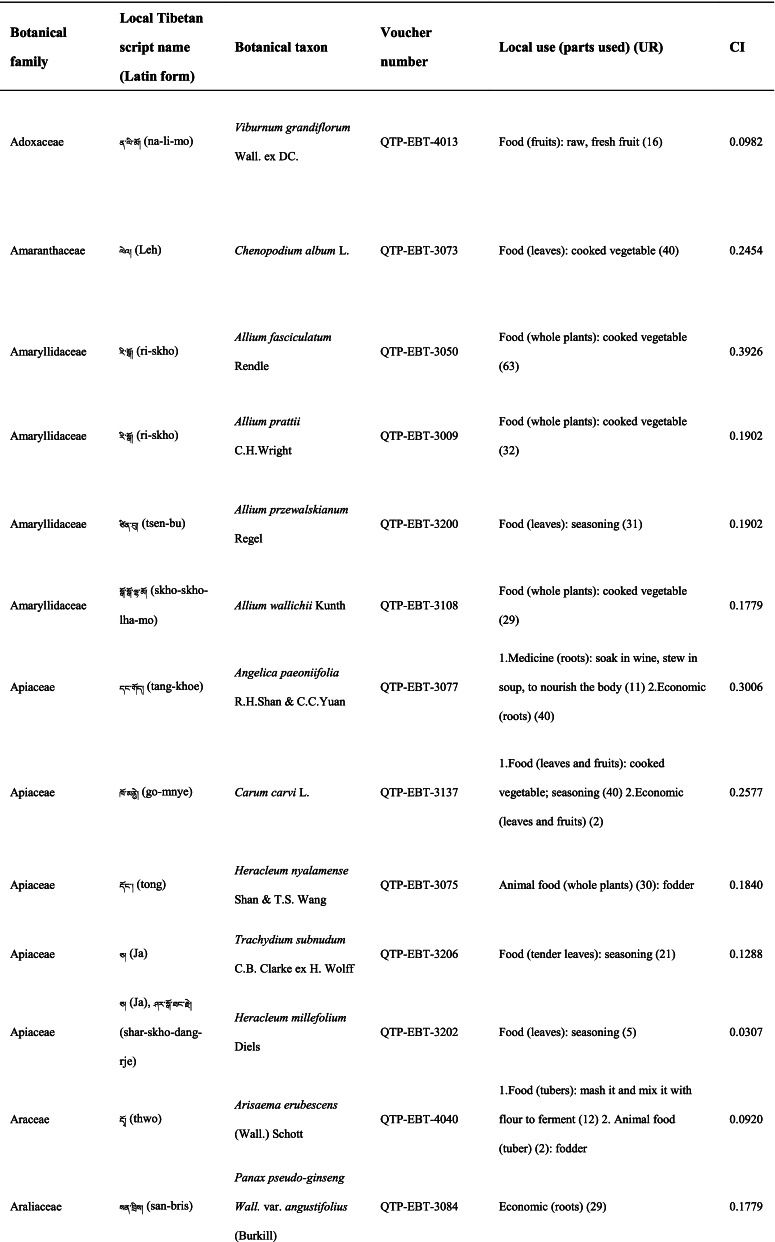

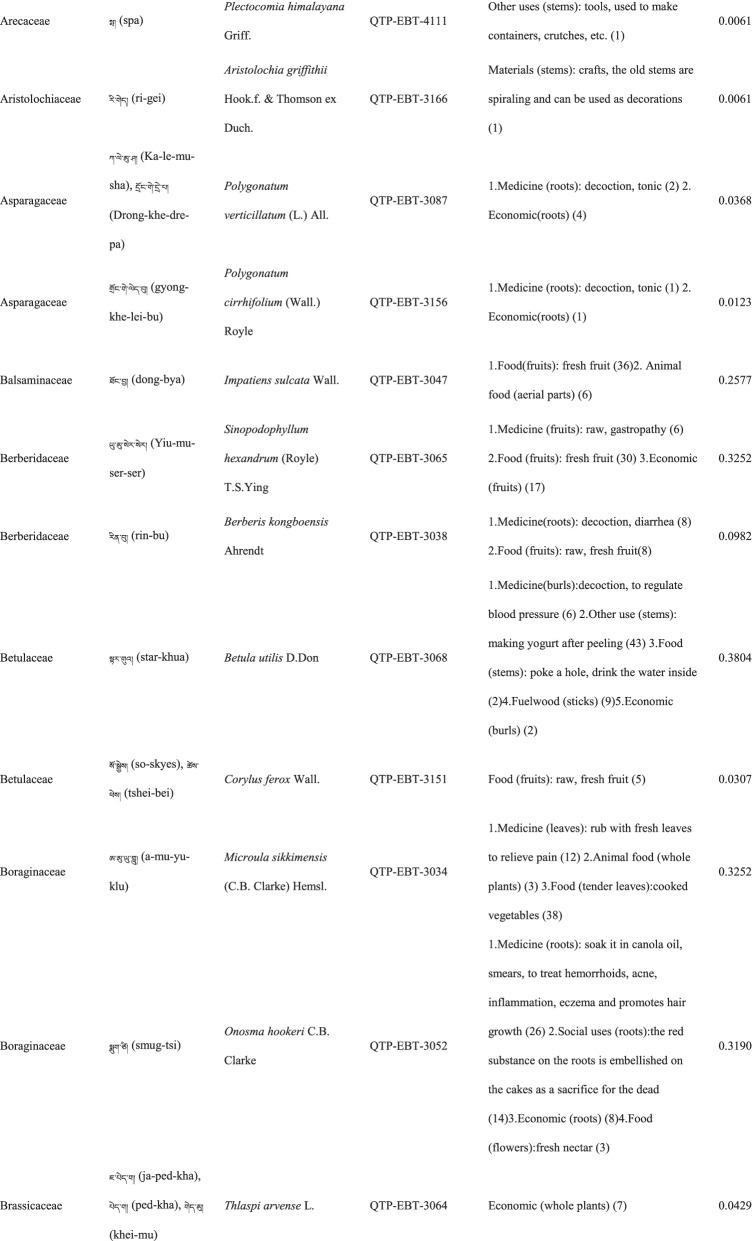

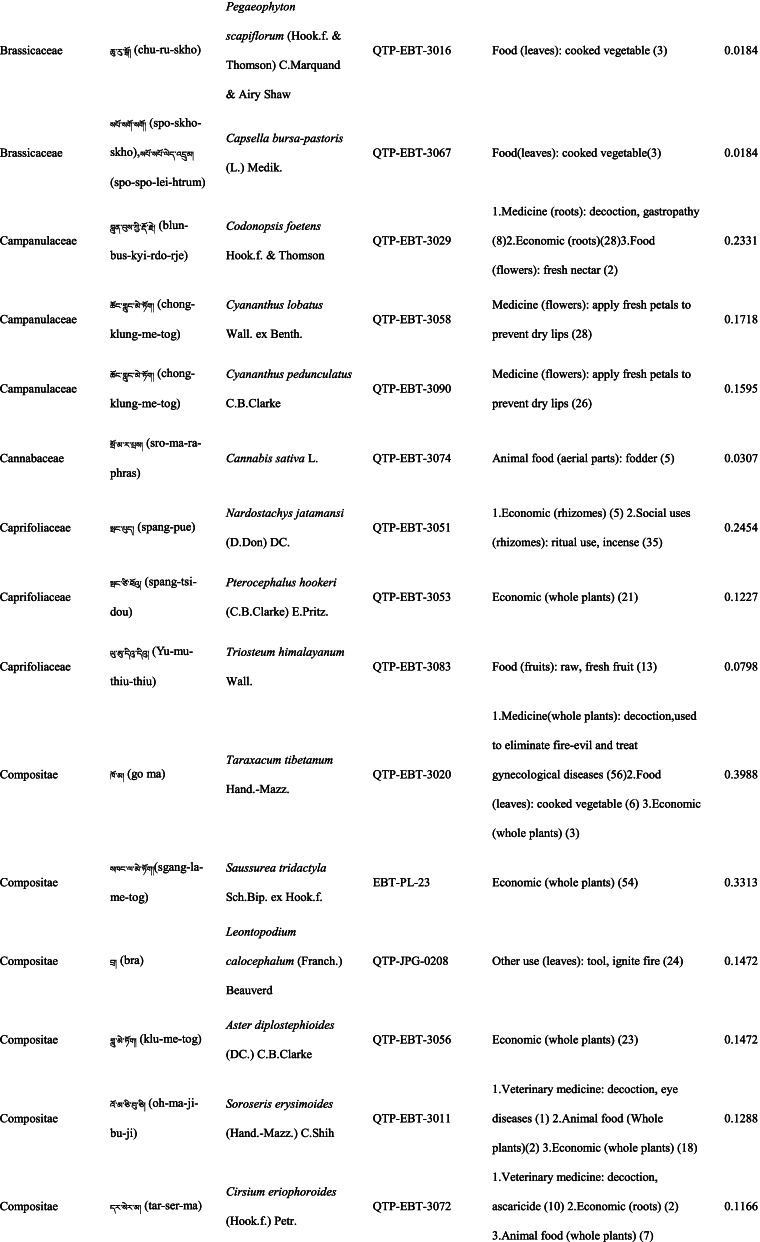

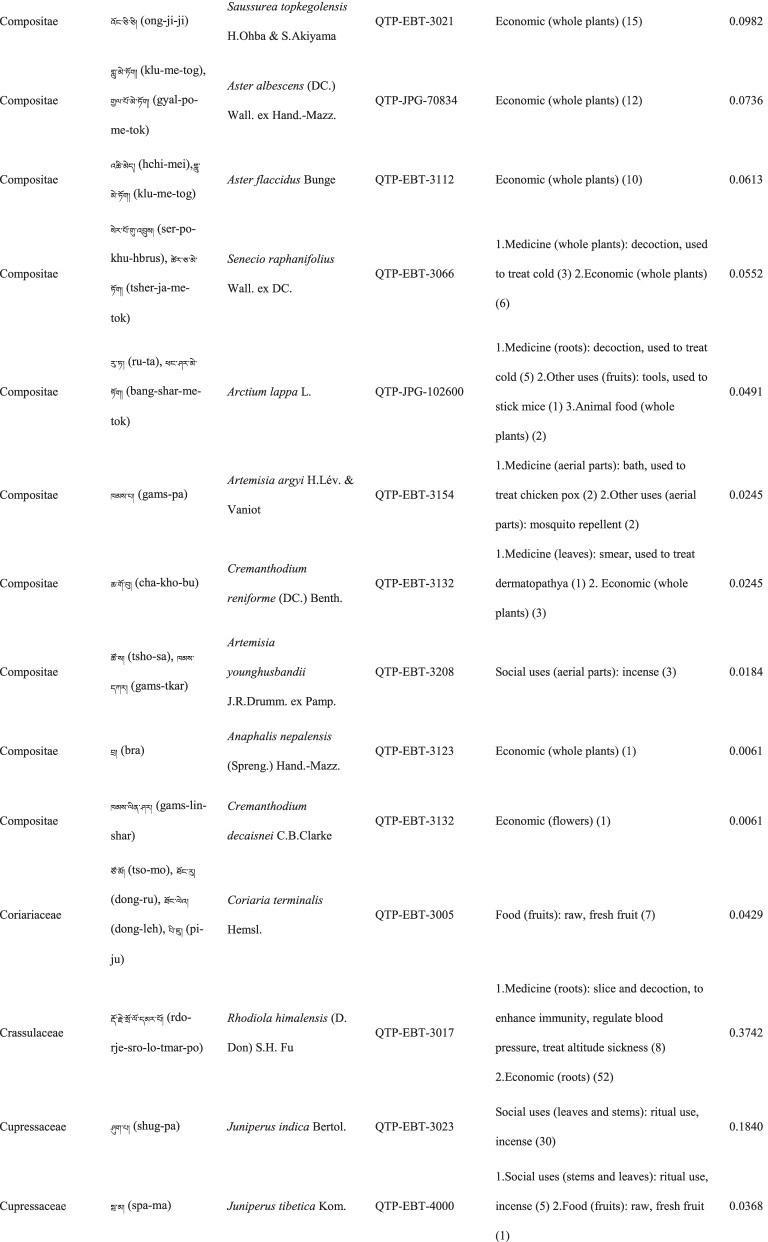

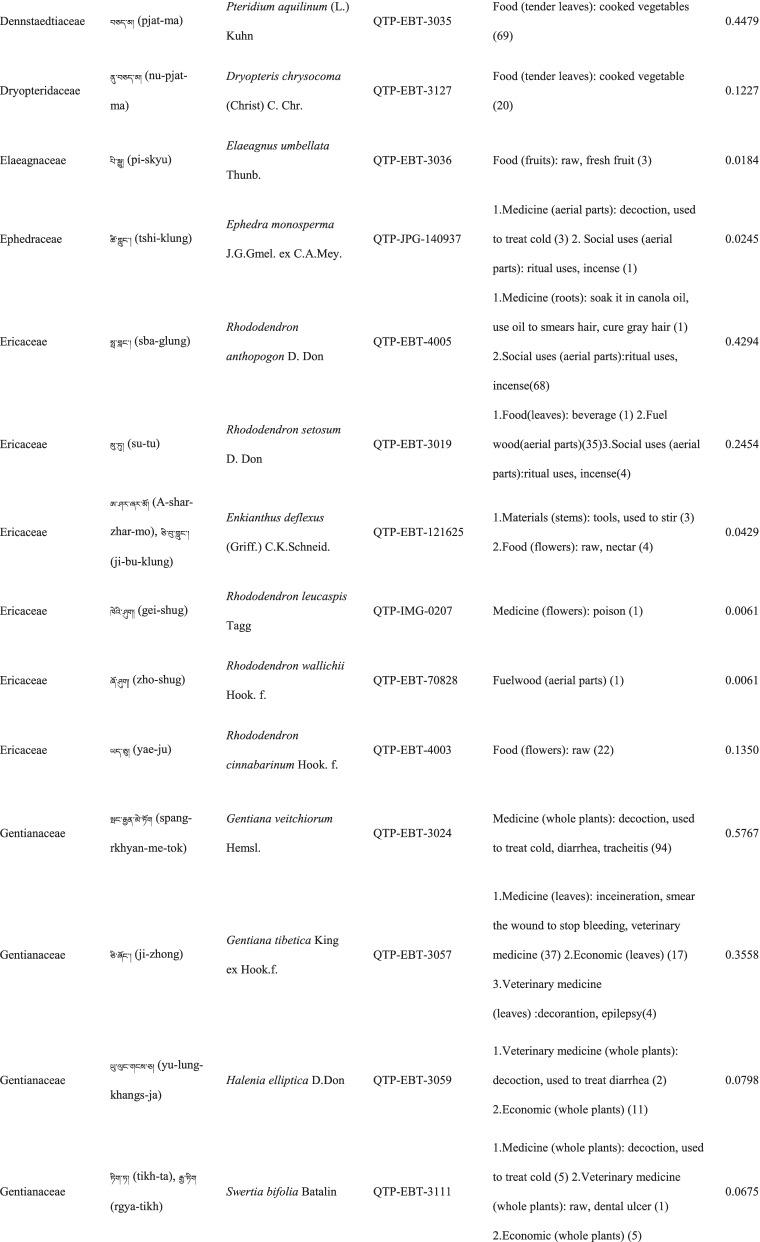

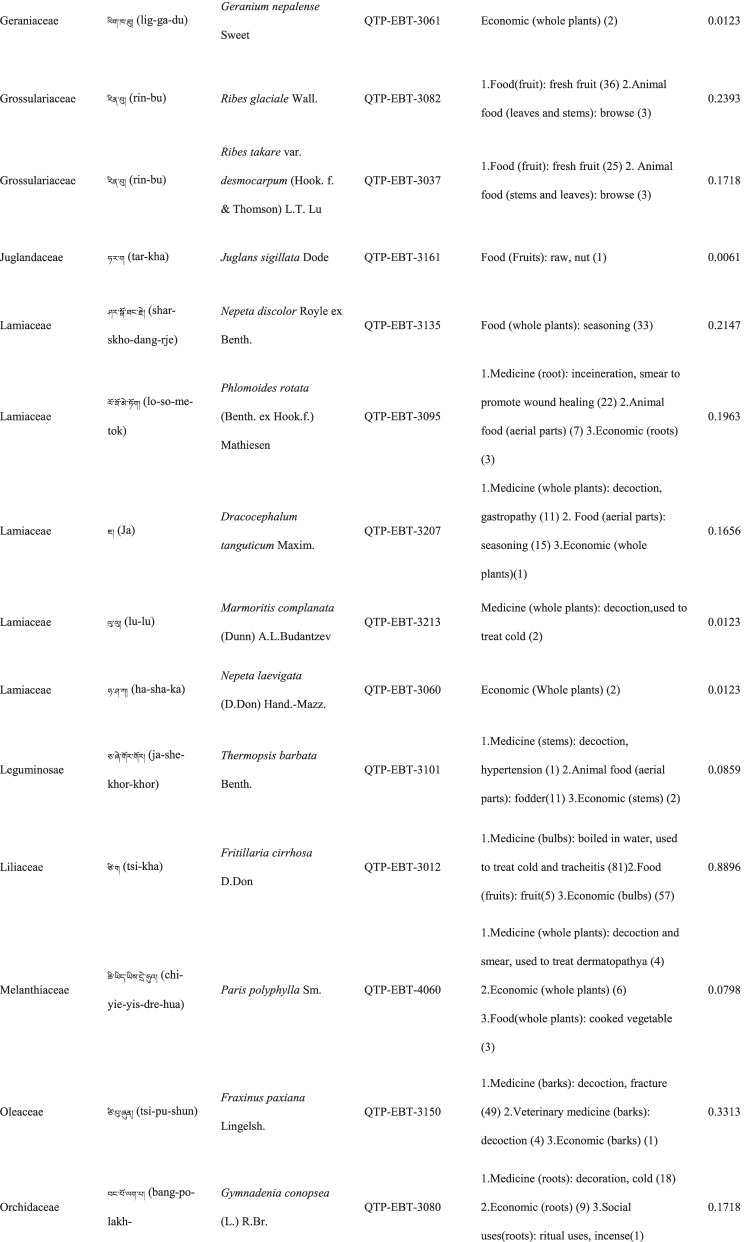

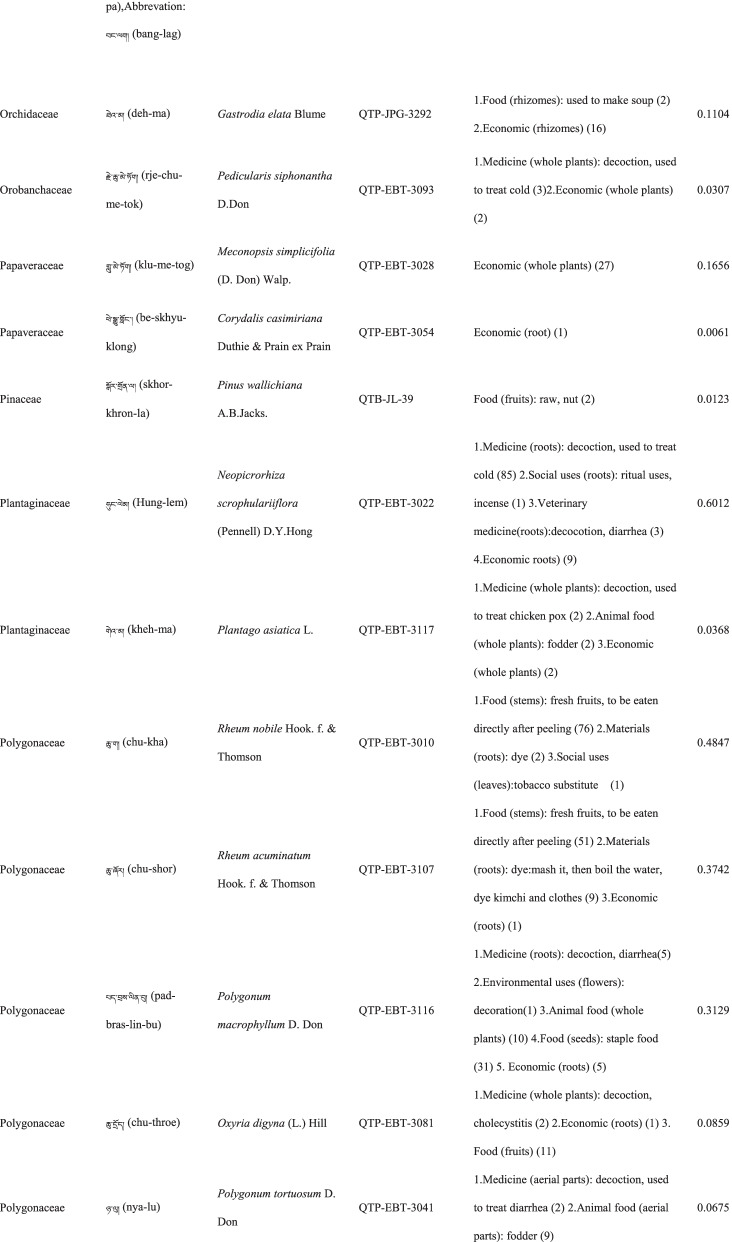

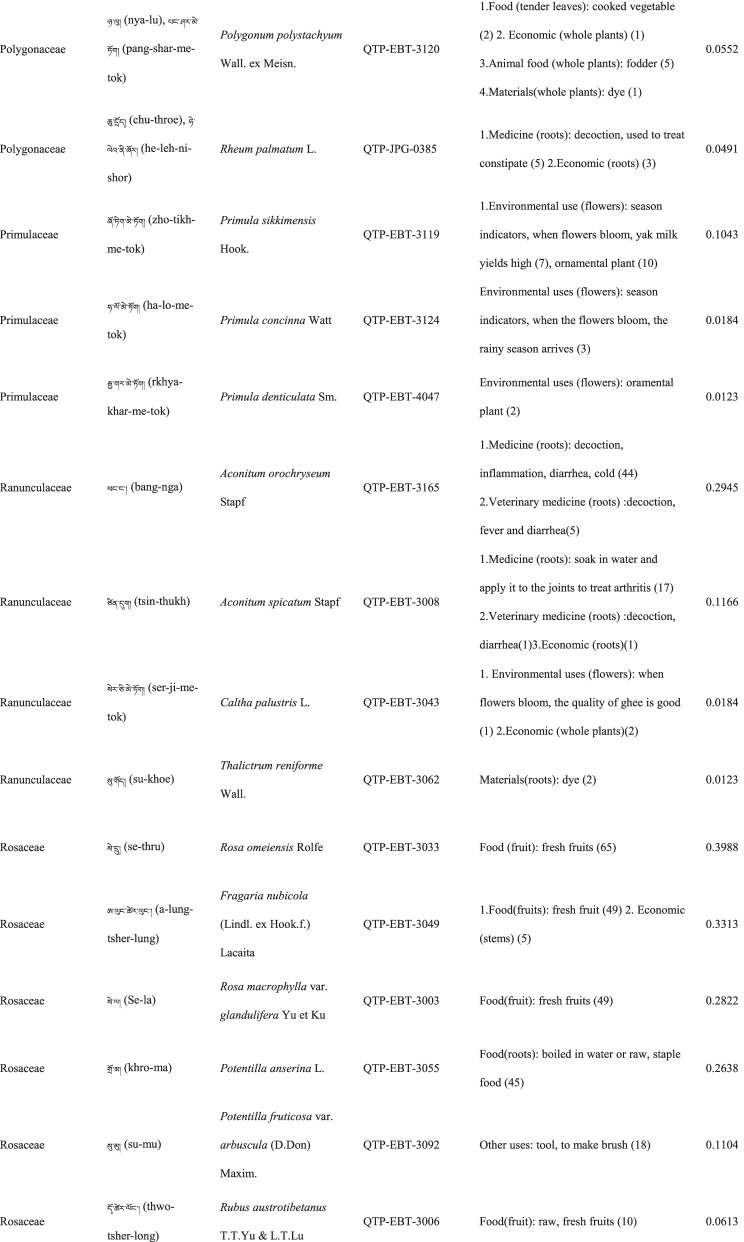

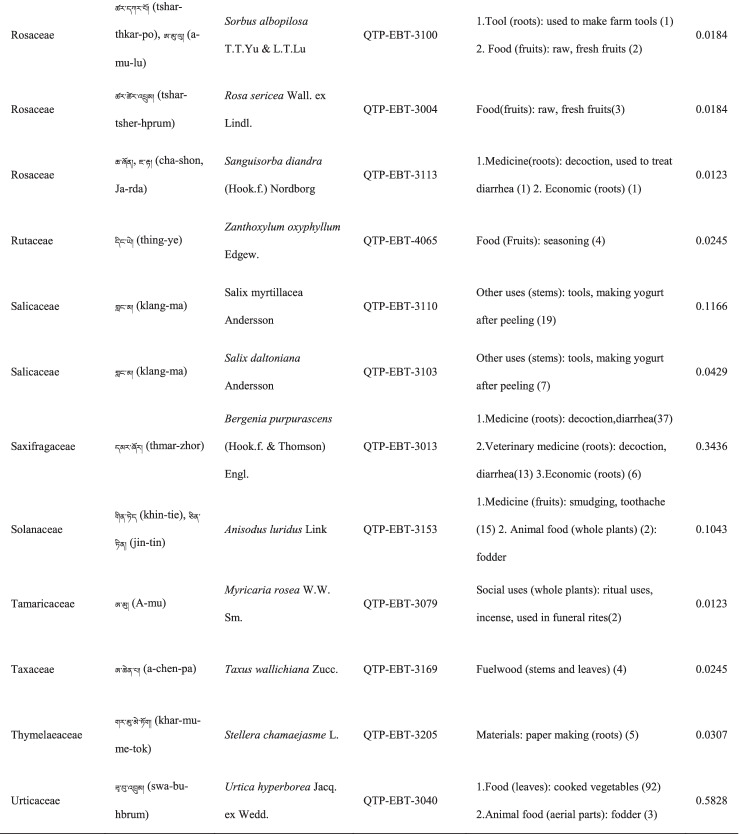
The wild plants used by the Yadong Tibetan

In our survey, the most frequently used parts were whole plants (37), followed by fruits (27), roots (27), leaves (23), stems (16), aerial parts (13), flowers (12), bulbs (1), seeds (1), and burls (1) (Table 2).

### The diversity of use categories

The informants referred to 3,028 URs, which 1,177 (38.8%) were for food uses, 805 (26.6%) for medicinal uses, 560 (18.5%) for economic plants and 486 (16.1%) for other categories. There were 53 edible plants species, 53 economic plants, 46 medicinal plants, and 50 plants used in other categories, including animal feed (18), social uses (12), environmental uses (6), materials (5), fuel (4), and other uses (10) (Table 3). Many plants belonged to multiple utilisation categories. There were 30 plant types plants with three or more uses: 12 medicinal and edible homologous plants, 32 plants that could be used both as a source of income and as medicinal plants, and 15 plants that could be used as both a source of income and food (Table 2).

**Table 3:** Informant consensus factor for traditional medicinal plant use categories

Secondary category of use	Tertiary category of use	Number of use reports(Nur)	Number of taxa (Nt)	Informant consensus index factor (FIC)
Cardiovascular disease	Hyperglycaemia, hypertension, anaemia	35	7	0.82
Dermatopathya	Burn, bleeding, acne	116	6	0.96
Gastrointestinal problems	Constipation, diarrhea,gastralgia	118	10	0.92
Infections	Chicken pox	4	2	0.67
Poisons	Poisons	1	1	–
Respiratory complaints	Cold, begma	321	11	0.97
Skeleto-muscular system	Fractures, arthralgia	66	2	0.98
Toothache and mouth	Toothache	15	1	1.00
	Chapped lips	54	2	0.98
Hypoimmunity	Hypoimmunity	2	1	1.00
Inflammations	Inflammations	142	4	0.98
Hair follicle	Promote hair growth, hair darkening	14	2	0.92
Cholecystitis	Cholecystitis	2	1	1.00
Veterinary medicinal	Ulcer, parasites, eyesache, fractures diarrhea	48	10	0.81

### Food

A total of 53 species of wild plants are used as food for humans. The food categories in Yadong include fruits (28 species), vegetables (16), seasoning (7), starches (3), and beverages (1) (Table 3). The most widely used component is the fruit. There are very few gardens and woodlands available for planting fruit trees in the local area, and the yield of local fruit tree varieties is extremely low. This may be one of the main reasons why the locals collect wild fruits from the wild as a nutritional supplement.

### Animal feed

Eighteen wild plant species were used as animal feed. Among these 18 species, 16 were herbs, and 2 were woody plants (Table 3). These included *Heracleum nyalamense* (CI_animal food_ = 0.1840), *Thermopsis barbata* (0.0798), *Polygonum macrophyllum* (0.0613), *P. tortuosum* (0.0552), and *Cirsium eriophoroides* (0.0429). *H. Nyalamense* is the most popular animal feed plant for locals. Local people indicated that cattle grew more strongly after feeding on this grass.

### Medicinal plant use

A total of 43 traditional medicinal plants belonging to 24 families and 39 genera have been documented to treat 14 different types of human diseases, including dermatologic disorders, gastrointestinal problems, respiratory diseases, diarrhoea, and arthritis (Table 4). The most cited families of medicinal plants were Compositae (7 species), followed by Polygonaceae (4), Gentianaceae (4), and Lamiaceae (3). The plant parts most commonly used for remedy preparation are the roots accounting for 45.5% of the total medicinal plants. In addition, 10 veterinary medicines were used to treat 5 types of animal diseases (Table 4). The five most-cited species were *Gentiana veitchiorum* (CI_medicine_ = 0.5767), *Neopicrorhiza scrophulariiflora* (0.5215), *Fritillaria cirrhosa* (0.4969), *Taraxacum tibetanum* (0.3436), and *Fraxinus paxiana* (0.3006) (Table 2).

**Table 4:** Use categories and use reports

The first category	The second category	Criteria	No. of species	Use reports
Food	Fruits	Fruits that were only eaten when they were ripe, such as apple, pear, strawberry	28	534
	Vegetable	Plants material what were used to cook dishes (including making salads directly with raw plant material	15	408
	Seasoning	Plants that could be added to dishes or soups to increase the flavour of food	7	148
	Starches	Plants that could be used as a direct starch supplement (e.g., tuberous or rhizome of some plants) or processed into starch	3	86
	Beverages	Plants that could be processed into homemade liqueurs or alcoholic beverages and processed into herbal teas	1	1
Economic plant		The living plant, plant part, or derived product that can be traded	53	560
Medicine	Medicine for human	Plants that could be used by local people to treat diseases of human	43	761
	Veterinary medicine	Plants that could be used by local people to treat diseases of animals	10	44
Animal feed	Fodder	Food (herb) for horses and farm animals	16	107
	Browse	Food (leaves of wooden plants) for horses and farm animals	2	6
Social uses	Ritual uses	Plants used in social scenarios, such as incense	11	165
	Somoking substitute	Plants that are substitutes for tobacco	1	1
Fuel	Fuelwood	Wood used for fuel	4	49
Materials	Dyes	Plants that can be used to dye something	3	13
	Crafts	Plants for making crafts, such as wooden bowls	1	1
	Paper making	Raw materials for papermaking	1	1
Environmental uses	Oranmentals	Plants that can be used for ornamental purposes, such as potted plants, headdresses	3	13
	Season indicators	Plants that can indicate the arrival of the season	3	11
Other uses	Tools	Plants that can be used to make tools, such as containers, cookware	9	117
	Repellent	Plants used to repel mosquitoes	1	2

The FIC of 14 diseases ranged from 0.67 to 1, and were the highest for toothaches (1.00) and hypoimmunity (1.00), followed by inflammation (0.98), skeleto-muscular system disorders (0.98) , and respiratory complaints (0.97). The FIC values were lowest for infections (0.67). The most cited diseases were respiratory complaints (321 URs), followed by inflammation (142), gastrointestinal (118) and dermatopathya (116) (Table 4).

### Economic plants

Yadong County is rich in medicinal and non-timber forest products. We found 53 commercial plants in the study area. These plants were mainly sold to Tibetan doctors and Hui merchants. Among them, 32 species were used as medicinal plants by locals (Table 3). The most frequently mentioned economically important plant was *Fritillaria cirrhosa* (CI_economic_ = 0.3374), followed by *Saussurea tridactyla* (0.3313), *Rhodiola himalensis* (0.3252), *Angelica paeoniifolia* (0.2454), and *Panax pseudoginseng* (0.1779) (Table 2). In addition, local people also collect *Cordyceps sinensis* or *Exidia* sp. as important sources of income.

**Fig. 3:**
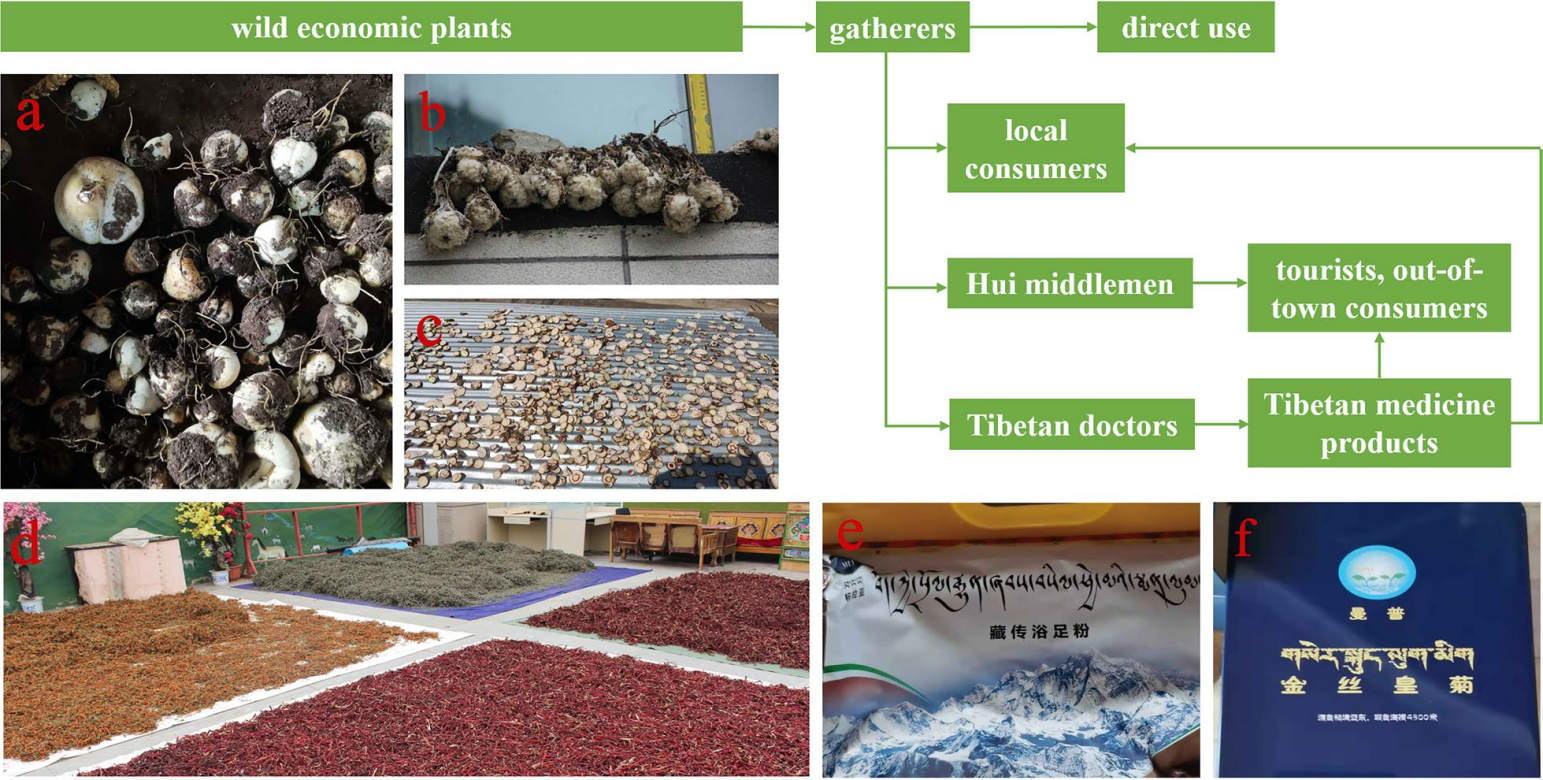
Several uses of economic plants from collection to utilisation. **a** *Fritillaria cirrhosa* D. Don. **b** *Saussurea tridactyla* Sch. Bip. ex Hook. f. **c** *Rhodiola himalensis* (D. Don) S.H. Fu. **d** Locals drying herbs in the garden. **e, f** Tibetan medicine products

In the local region, except for a small number of wild economic plants that collectors directly use, most plants enter the local or foreign market in somehow (Fig. 3).

### Social uses

Social uses were divided into two categories: ritual plants (11 species), and tobacco substitutes (1) (Table 3). Tibetan people convey their wishes to the gods through various sacrificial activities and offer them several items, praying for happiness and well-being. A total of 11 species of plants are used in social activities. Among them, nine species were used to incense. For example, *Rhododendron anthopogon* (CI_Social uses_ = 0.4417), *Nardostachys jatamansi* (0.2147), and *Juniperus indica* (0.1840). Two species were used at funerals. The local people use the purple pigment from *Onosma hookeri* (0.0675) roots to decorate the offerings. After a person dies, *Myricaria rosea* (0.0123) sticks are burned to pay homage to them.

### Environmental uses

In the local region, milk collection showed seasonal characteristics and wild plants can be used for climate prediction [55]. Three plant species were mentioned in the interviews; *Primula sikkimensis* was the most frequently mentioned, followed by *P. concinna* and *Caltha palustris*. The yield and quality of yak milk are high when *P. sikkimensis* and *C. palustris* are in bloom The flowering of *P. concinna* heralds the arrival of the rainy season and is also the time for planting and grazing. These plants have some common characteristics. These plants tending to grow in around pastures and farmlands. In addition, flowering was considered a climate predictor by most respondents.

### Materials and other uses

Five plant types are used as raw materials for dyeing (3), papermaking (1), and crafts (1) (Table 3). However, the frequency of mentioning this type of utilisation was very low, which may indicate that the local area is losing traditional handicraft knowledge or that this knowledge is in the hands of only a few people. *Rheum acuminatum*, *Rheum nobile*, and *Polygonum tortuosum* are used in traditional dyeing. *R. acuminatum* and *R. nobile* roots are crushed, boiled in water, and used as yellow dyes. *Stellera chamaejasme* is used to make paper, which is insect repellent, antiseptic, and flexible. The old stems of *Aristolochia griffithii* were picked up by locals and polished into ornaments or stools.

**Fig. 4:**
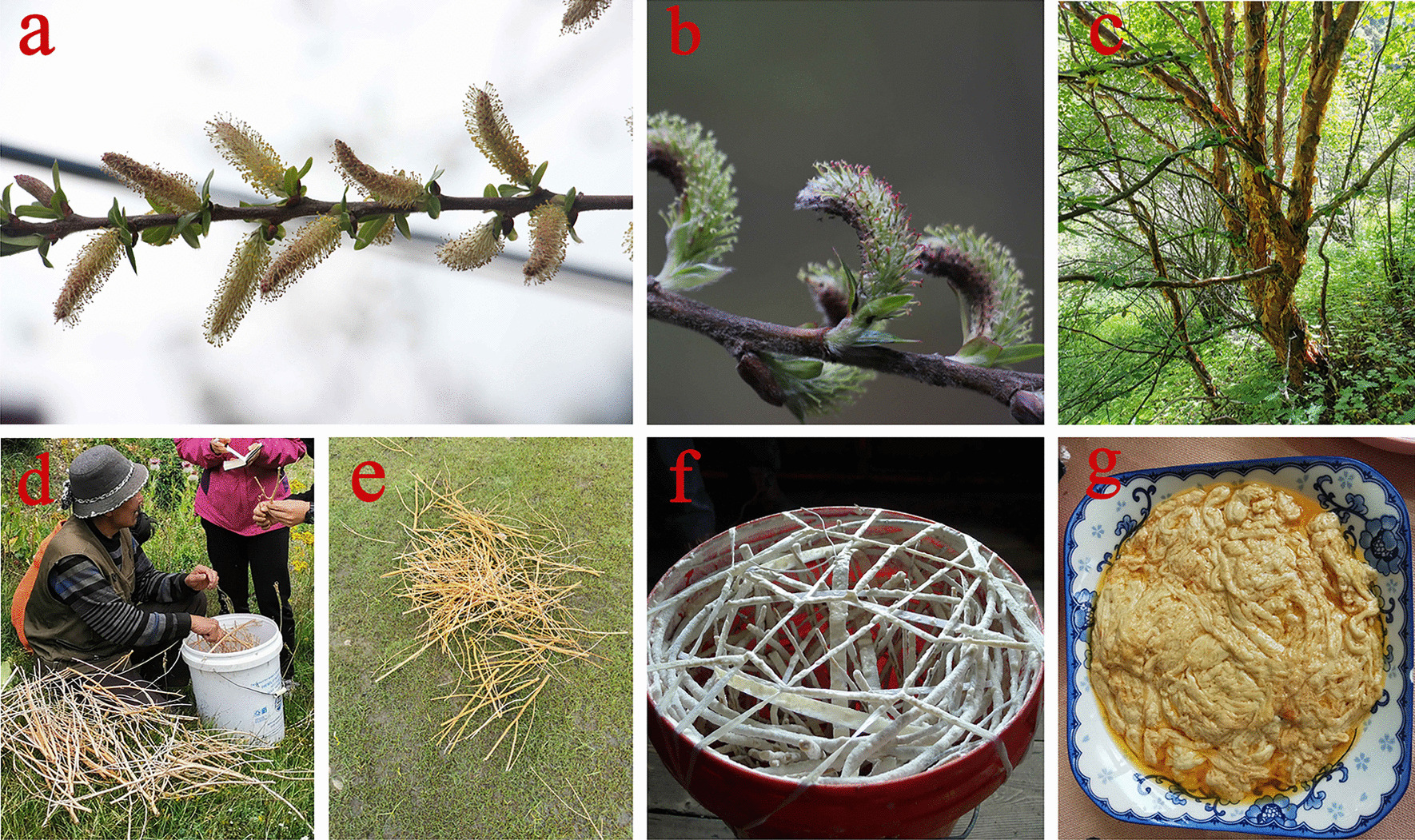
Tibetan cheese fermentation process. **a** *Salix daltoniana* Andersson. **b** *Salix myrtillacea* Andersson **c** *Betula utilis* D. Don. **d** Soak branches in boiling water, wash, and peel. **e** Cleaned branches. **f** Set up the branches in the bucket, pour the milk, shake the bucket to make the milk adhere to branches. **g** Put the fermented cheese into the pot, add ghee and fry until it is cooked

A total of 10 kinds of plants were used for other purposes (9 tools, and 1 repellent). Five plant species were used as make cooking tools (Table 3). For example, *Betula utilis* sticks can be used to make spoons or shovels. *Potentilla fruticosa* var. *arbuscula* sticks are used to make brooms for washing pots. *Enkianthus deflexus* branches are used to make a blender, which is used to prepare milk tea. Local Tibetans prefer to eat a fermented milk product (*pilu*), which has a unique taste, and whose fermentation process is notable. The locals collect *Betula utilis*, *Salix myrtillacea*, and *Salix daltoniana* branches, boil, peel, and place them in a bucket, after which raw milk is poured over them and sealed to prevent mosquitoes from entering. This bucket is rotated and shaken daily so that raw milk is evenly attached to the branches. After 15 days, the local speciality food *pilu* (smelly cheese) is produced. *Pilu* must be cooked with yak butter before it can be eaten (Fig. 4). In the process of producing *pilu*, there is a critical step: placing branches in the bucket. If branches are not added, water loss will be slow and the milk will spoil. When asked why they chose the three trees mentioned above, the locals gave the following answers: they were easier to obtain, nontoxic and easier to peel their bark; most importantly, their branches were not easily corroded and could be reused many times.

Despite of social changes, some crafts and tools are still handmade and use plant materials [56]. These plants used to be closely related to the daily life of local people, but now they provide more cultural service.

## Discussion

### Current situation of the traditional knowledge of plant use

Local Tibetans have a wealth of knowledge of plant utilization, which penetrates into all aspects of live. But these knowledge was mainly distributed among middle-aged people and is severely affected by socio-economic development.

Tibetans have unique dietary habit under special geographical and climatic conditions. Highland barley powder and ghee are the main foods of farmers and herders, and are supplemented by dairy products such as yoghurt and milk residue, as well as meat of mostly beef and lamb [57]. Fruits and vegetables are rare, but in Yadong, many wild vegetables and fruits are used, and these account for 80% of wild edible plants. With the increasing demand for medicinal plants in Tibetan traditional medicine and Chinese medicine, the commercial value of medicinal plants has increased. Yadong County is rich in medicinal plant resources; thus, economic utilisation has become an important category of plant utilisation. In addition, animal husbandry is the main source of livelihood for the local people, and fermented yoghurt plants, animal feed, and seasoning plants related to animal husbandry are also frequently used in Yadong. Herders are the main collectors of high-altitude medicinal plants, which are harvested from alpine meadows and pastures, consistent with ethnobotanical studies in Dolpa, Humla, Jumla, and Mustang districts of Nepal [27].

Experience is an important means for transmitting traditional knowledge [3]. In the past, living conditions were relatively poor, and young people often followed their elders to gather food, medicine, fuelwood, etc. in the wild. However, the current rapid economic development has resulted in significant supplementation of material resources, and the collection of wild plants has become less necessary. More children are now going to boarding schools in the county and to more developed areas to make a living. Most young people are not interested in traditional medicine, and ethnobotanical studies in some parts of Nepal have described the same phenomenon [28, 58]. Elderly individuals may slowly lose their memory of wild plant use because of socio-economic changes over the years. These reasons have resulted in obstacles to the inheritance of traditional knowledge, and information that has been passed down from generation to generation has become distorted [23, 24]. Ultimately, modern lifestyle changes are the main reason for the decline in traditional plant knowledge [41].

### The most popular edible plants

Wild edible plants (WEPs) play an important role in food supplementation under normal circumstances [58] and are an important source, in addition to cultivated plants, for people to obtain nutrients, vitamins, minerals, and other biologically active compounds [48, 59].

Fruits with high CI values included *Rheum nobile* (CI_fruit_ = 0.4663), *Rosa omeiensis* (0.3988), and *Fragaria nubicola* (0.3006). The tender stems of *R. nobile* are peeled and eaten raw as snacks by locals. The plant is mainly distributed in the local high mountains at over 4,000 m above sea level [48]. Plants of the same genus ar abundant in other Tibetan regions. Tibetans from Lithang eat *R. alexandrae* and *R. palmatum* stems after removing the skin [36]. Tibetans eat the tender stems of *R. officinale* and *R. palmatum* in Zagana, Gansu, China [37]. The Tibetans of Shangri-La, Yunnan, China eat the tender leaves of raw *R. likiangense* [32]. In the Ladakh region, locals eat the young stems of *R. webbianum* [60]. The ripe fruits of *R. omeiensis, R. macrophylla* var. *glanduslifera and R. sericea* are enjoyed by locals, especially children. *R. omeiensis* is also eaten by Tibetans in Sichuang, Gansu, and Shangri-La, Yunnan, China [32, 36, 37]*.* In addition, the Lhoba people in Douyu village, southeastern Tibet, use it as a medicinal plant to treat anaemia and maintain youth [61]. *F. nubicola* can be eaten raw or made into jams, or eaten with shaved ice. In Nepal Tibetans of the Mustang District also eat this plant as a fresh fruit [36]. Very few gardens and woodlands are available for planting fruit trees in the local area, and the yield of local fruit tree varieties is extremely low. This may be one of the main reasons locals collect wild fruits as a nutritional supplement.

*Urtica hyperborea* (CI_vegetable_ = 0.5644) and *Pteridium aquilinum* (0.4294) had vegetables with high CI values. The tender leaves of *U. hyperborea* are locally eaten as wild vegetables. It is consumed in spring and stored as a reserve vegetable during winter. Boiling with rice or tsampa is the main processing method by which locals detoxify the plant. The tender stems of this plant are used to stew soup by Tibetans in Sapi, Ladakh, Jammu and Kashmir, India [36]. A previous study showed that *U. hyperborea* extract lower uric acid levels [62]. Hyperuricaemia and gout affect humans globally [63]. There are abundant resources of *U. hyperborea* in China [64]. Therefore, *U. hyperborea* is expected to be developed into a healthy food source in plateau areas. Young leaves of *P. aquilinum* were collected from the local population; these were blanched and soaked overnight after which they can be cooked as seasonal vegetables. Locals indicated that soaking overnight removes the bitterness and to improves the taste of young leaves. However, this plant contains the toxic compound ptaquiloside (PT), which is carcinogenic [65]. Fortunately, the soaking process removes the carcinogenic toxic substances present in this plant [66]. *P. aquilinum* is also eaten as a wild vegetable by Tibetans in Zhagana, Gansu, China and Shangri-La, Yunnan, China [32, 37]. In addition, *P. aquilinum* has a high commercial value in Gongba, Gansu, China [35].

The two most popular seasoning species were *Carum carvi* (CI_seasoning_ = 0.2270) and *Nepeta discolor* (0.2147). *C. carvi,* also called “kuo nie”, is the most frequently mentioned spice in Yadong. Local Tibetans collect its young leaves in May or June and fruit in August or September. The fruits of *C. carvi* have a pungent, coriander-like flavour and aroma that originates from essential oils, mostly carvone, limonene, and anethole [67, 68]. *C. carvi* is a source of cumin and caraway seeds, which have been used since ancient times to treat various indications in traditional healing systems in wide geographical areas [69]. Since ancient times, *C. carvi* has been used in Europe as a seasoning spice and an aromatic repellent. It can not only improve eyesight but also make the breath more fragrant. The seeds of this plant are also used as seasoning by Tibetans in Lithang, Sichuan, China and Mustang, Nepal [36]. In addition, the seeds of *C. carvi* is also used a cure for poisoning and fever, promote appetite, and improve digestive health in Lithang, Sichuan, China [70]. The local people collect the aboveground parts of *N. discolor*, and eat them as condiments after drying in the shade.

The locals identified three important starch supplement plants, *Potentilla anserina* (CI_starch_ = 0.2638), *Polygonum macrophyllum* (0.1902). *P. anserina* (Chuoma) is frequently used as a staple in modern Tibet. *P. anserina* roots are boiled and eaten with butter and sugar. This traditional dish is served on important Tibetan holidays [35, 37]. Compared with traditional root foods such as *Solanum tuberosum*, *Ipomoea batatas*, and *Colocasia esculenta*, the ratio of nutrients (protein 9.45%, fat 1.15%, dietary fibre 15.23%) in this plant is healthier and more reasonable [71]. *P. anserina* is also eaten as a substitute for tsampa by the Tibetans Litang in Sichuan, Zhagana in Gansu, and Shangri-La in Yunnan, China [32, 36, 37]. In addition, the local population mix *P. macrophyllum* and *Hordeum vulgare* var. *coeleste* seeds and grind them into flour. Tibetans in Zhagana, Gansu, China use them similarly [37]. Wild starch plants have become an important source of supplementary starches.

These wild plants compensate for the lack of vegetables and fruits in the traditional Tibetan diet and the lack of starch in times of food shortages. In addition, the utilization of these plants also has a high correlation with the surrounding area, which also shows the transmission of culture.

### The most popular medicinal plants

We conducted a quantitative analysis based on the information provided by the participants. The five most-cited species were *Gentiana veitchiorum* (CI_medicine_ = 0.5767), *Neopicrorhiza scrophulariiflora* (0.5215), *Fritillaria cirrhosa* (0.4969), *Taraxacum tibetanum* (0.3436), and *Fraxinus paxiana* (0.3006) (Table 2)*.* As a part of the cultural diversity of Tibetan community, these traditional medicinal knowledge and experiences provided important health services for locals.

**Fig. 5:**
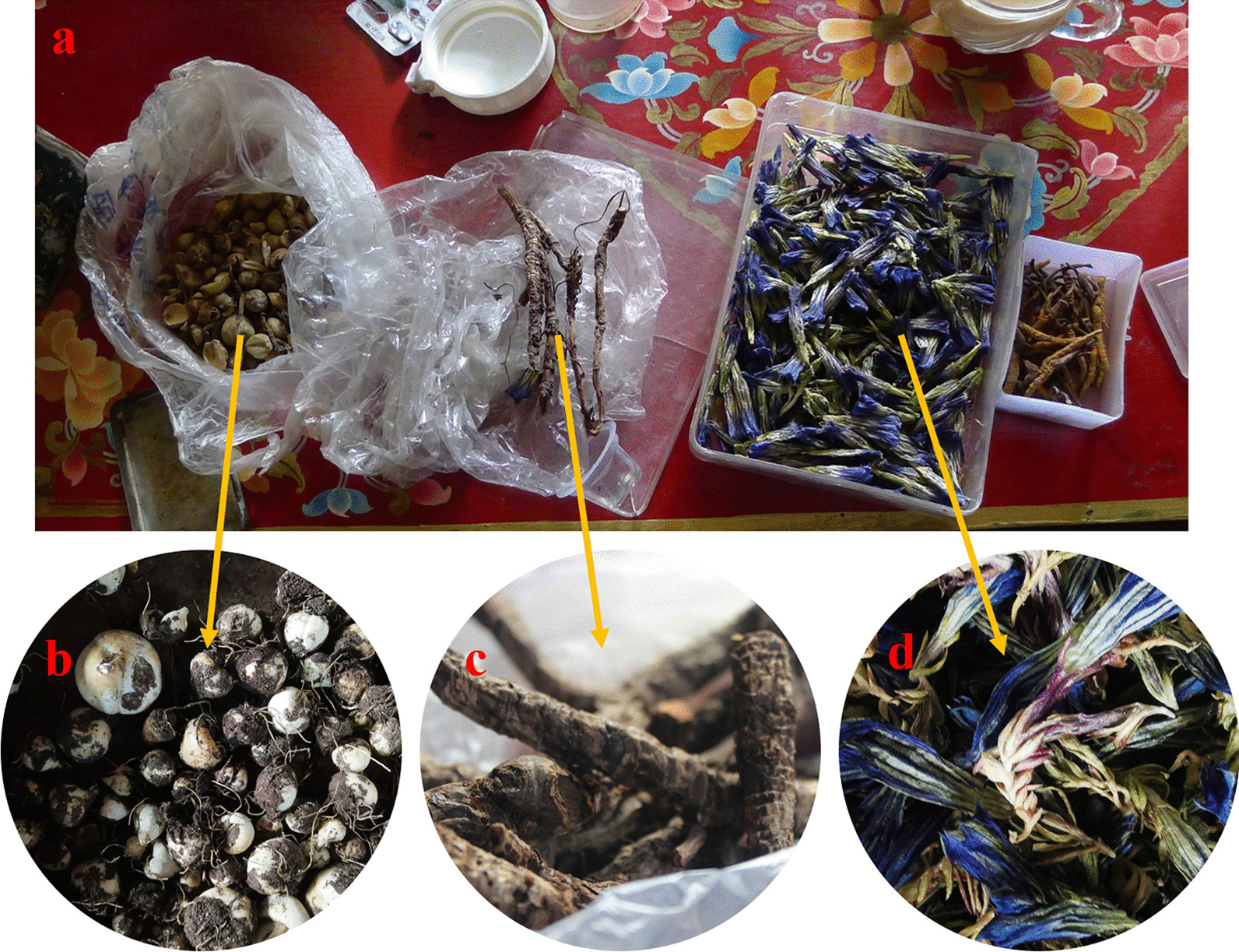
The top three medicinal plants. **a** Medicines stored in the home. **b** *Fritillaria cirrhosa* D.Don. **c** *Neopicrorhiza scrophulariiflora* (Pennell) D.Y.Hong. **d** *Gentiana veitchiorum* Hemsl

It is worth noting that the three most cited medicinal plants are all used to treat respiratory diseases and are usually stocked in the homes of locals to meet daily needs, much like a medical kit for city family (Fig. 5a). Respiratory diseases are the most common diseases locally, which is consistent with ethnopharmacological survey data in Dolpa, Humla, Jumla, and Mustang districts of Nepal [27]. These plant species are all were traditional Tibetan medicines [40, 41] and are used to treat common ailments, such as inflammations, colds, coughs, and diarrhoea. The whole plant body of *G. veitchiorum* (Fig. 5d) is used locally to treat colds and bronchitis, and relevant studies have shown that the plant species have antibacterial, antiviral, and pharmacological activities for treating bronchitis [72–74]. The roots of *N. scrophulariiflora* are widely used by the locals to treat cold (Fig. 5c). According to the Chinese Pharmacopoeia, this plant can treat many diseases [44]. However, there is no documented treatment for the common cold and use as a veterinary medicine. In the Maithili region of eastern Nepal, the plant is used by locals to treat fever and headaches with high consensus [30]. The bulbs of *F. cirrhosa *(Fig. 5b) are used by the locals as a medicine to treat tracheitis, and this species is also rich in pharmacologically active compounds with antitussive activity [75, 76].

*T. tibetanum* whole plant is added to boiling drinking water, and to treat various inflammations, such as upper respiratory tract infections, and pharyngitis. Young leaves can be collected and used as wild vegetables with a bitter taste. Locals indicated that this vegetable could “clear the heat and remove the fire” and act as a nutritional supplement. Plants of the same genus are also used by Sherpas in Chentang, China, as medicines for cancer and gynaecological diseases, and Tibetans in Shangri-La, China, and Nepal also use it as a wild vegetable [32, 36]. The dandelion plant has a variety of active anti-inflammatory ingredients and contains various nutrients, such as proteins, sugars, vitamins, required by the human body. It is a medicinal and food homologous plant with great developmental value [77].

**Fig. 6:**
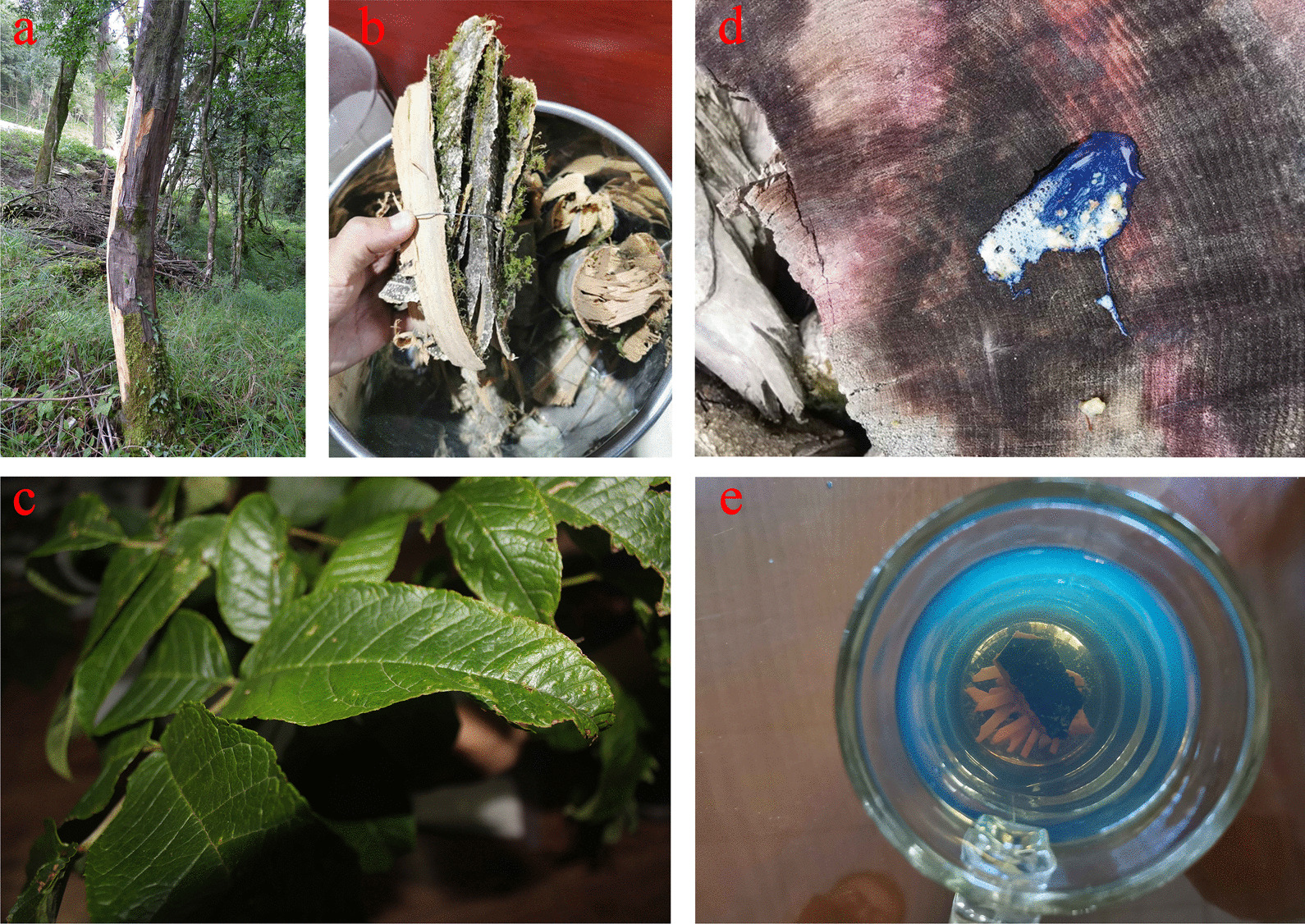
The process from collection to utilisation of *Fraxinus paxiana* Lingelsh. **a** the tree. **b** barks. **c** leaves. **d, e** The bark turns the water blue

The bark of *F. paxiana* can be soaked in water to treat fractures and can be used by both humans and livestock. In addition, during the collection process, the locals maintain part of the bark to ensure the nutrient supply of the tree itself, which is also an important manifestation of sustainable collection (Fig. 6). The same usage has been observed in Bhutan [78]. People in Nepal use plants of the same genus to treat body aches [79]. *F. paxiana* is mainly distributed in the subtropical rainforest below 2,000 m above sea level in the Yadong River Valley, where the population is very small. The main source of *F. paxiana* is Bhutan, where the private sector exchanges and purchases good through trade channels. Local merchants buy the bark of the plant from Bhutan and supply it to drugstores for trading. Although the plant is widely used locally, it has not been documented in traditional Tibetan medicine, and there is no documentation of its efficacy in treating fractures in Chinese medicine [80]. We theorise that the traditional knowledge of this plant was obtained by locals in Bhutan' s trade and cultural exchanges.

### Economic plant collection and sustainable development

In recent years, Tibetan and Hui medicinal material merchants have collected a large number of medicinal plants in Yadong. The informants informed us that the collection of economic plants had become more intensive compared with approximately 10–20 years ago. However, with the development of commerce, the excessive collection of plants has caused a certain degree of damage to the local ecological environment [28, 34, 81]. The degree of collection and dependence on wild plants is related to the economic status of the local people. It is generally believed that when a certain plant has a high economic value, it may lead to the depletion of plant resources owing to excessive collection [82, 83]. For example, *Fritillaria cirrhosa*, which have high commercial value, has been excessively and indiscriminately harvested (Fig. 3). As a result, its resources are declining sharply, and it is on the verge of extinction [85].

While over-harvesting of some important medicinal plants has increased, many locals are working toward both biological conservation of the medicinal plants through sustainable harvesting and protection of wild species and conservation of their cultural heritage [34]. The local government is also aware of the impact of this uncontrolled gathering on natural vegetation. For example, locals have realized that the harvesting of *Rhodiola himalensis* leads to soil erosion, and the the harvesting of *Rhodiola himalensis* has been banned. *Rhododendron anthopogon*, *Angelica paeoniifolia* and *Fragaria nubicola*, *Rheum palmatum* are cultivated as commercial crops in Kangbu and Shangyadong Township. It is hoped that this will increase local revenues while reducing damage to natural resources.

Although there are many initiatives for sustainable collection and utilisation, these policies have not been fully implemented. As a result, the depletion of high-value medicinal plants that local livelihoods depend on has not been curbed [29].

### “*Sang*” in the daily life of local people

**Fig. 7:**
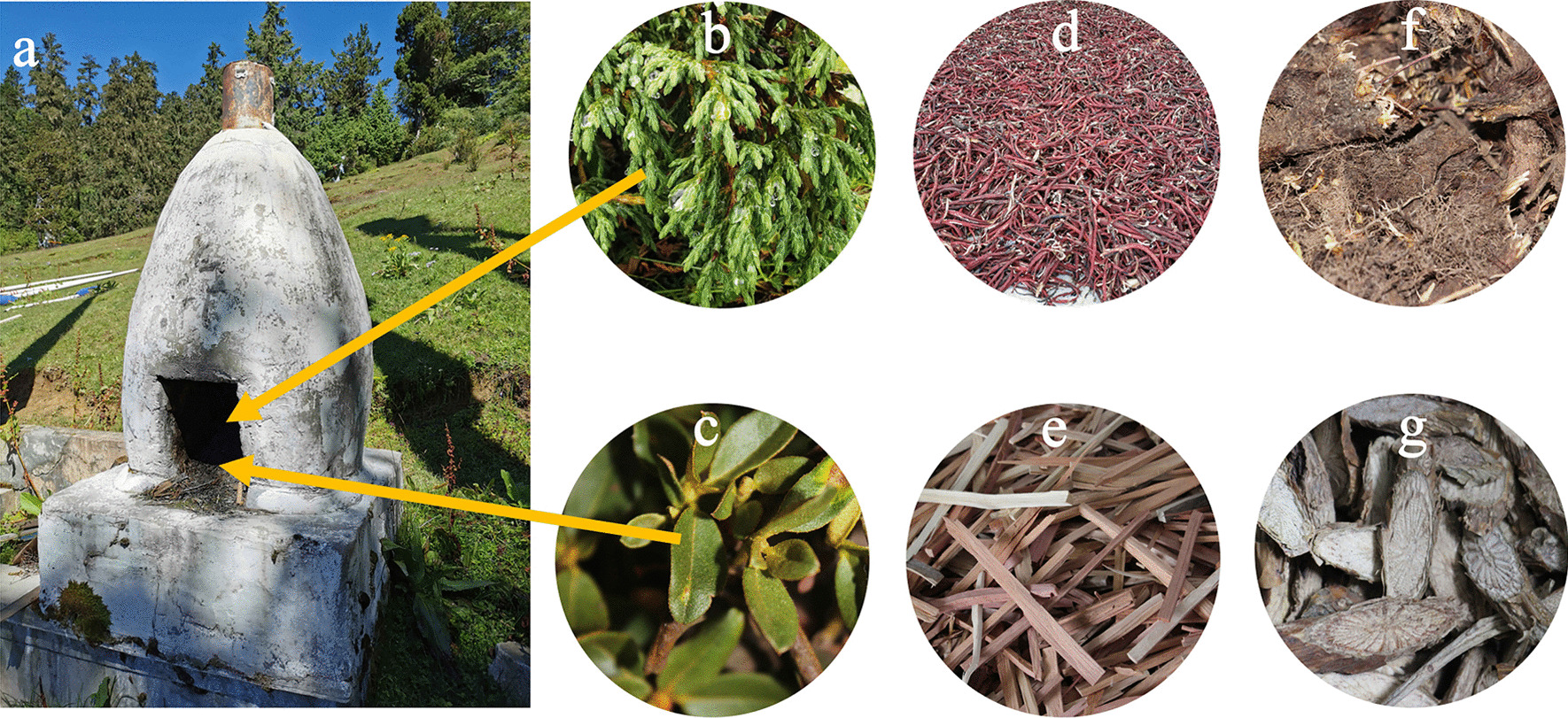
Some Tibetan incense plants and incense burner. **a** The incense burner, used to burn Tibetan incense plants. **b** *Juniperus indica* Bertol. **c** *Rhododendron anthopogon* D. Don. **d** *Onosma hookeri* C.B. Clarke. **e** *Juniperus tibetica* Kom. **f** *Nardostachys jatamansi* (D.Don) DC. **g** *Neopicrorhiza scrophulariiflora* (Pennell) D.Y.Hong

“*Sang*” is has important social uses and it prevails in Tibetan areas and has a long history [25, 85]. Most socially used plants are commonly used as incense materials, such as *Rhododendron anthopogon* and *Juniperus indica* (Fig. 7), and the dry sticks of these species are burned in a censer, which is placed on the flat roof of a house or at the entrance to the village. Local Tibetans in the Ladakh area also use *J. macropoda* as raw material for Tibetan incense [25]. These plants are usually burned in the early morning to pray for the gods’ blessing and good luck for the day.

In ancient society, when the men of the tribe returned after expeditions, hunting, or funerals, people thought that they were contaminated with all kinds of filth. Therefore, their family members used cypress branches and fireworks burned with various herbs to dispel the filth and to prevent its spread, which could, in turn, bring disaster to the family [85]. The following factors influence the plant preferences of local Tibetans. First, most of the plants burned in burner have a fragrance. After these plants are burned, they produce a strong fragrance. The scent drifts in all directions with the wind, so more gods can be reached, thereby fulfilling the needs of gods and providing better protection for the family. Second, most plants used for this ceremony are common people’s lives and surroundings.

In Tibetan areas, almost every household is equipped with a mulberry stove (or in the center of the yard, or on the roof by the mountain) (Fig. 7), and this ritual has become an important part of the local peopl’s live.

## Conclusion

This study demonstrates that the diversity of wild plants used by the Tibetan people in Yadong is reflected not only in the number of species but also in the diverse functions of wild plants, including edible plants, medicinal plants, animal feed, social uses, tools, dye, paper making, and other aspects. In this study, reports on the use of edible and medicinal plants were more prominent in the years when modern transportation was underdeveloped, and food supplies were insufficient. Locals have accumulated considerable experience in the use of wild edible plants, which s provide locals with a large amount of nutritional supplements and food supply.

With the development of the social economy, the demand for medicinal materials in the Tibetan and traditional Chinese medicine industries has increased, and the commercial value of many local medicinal plants has been identified. This has brought opportunities for local development, and has a negative impact on the environment. Locals are attempting to use artificial planting methods to reduce the hazards of overharvesting. Local traditional plant knowledge is also affected by the surrounding areas. This is likely because the Yadong River Valley has been an important trade channel since ancient times, where frequent cultural and trade exchanges have taken place.

In the future , more in-depth research should be conducted on the nutritional components and pharmacological activities of these plants. In addition, resource assessments of local plants with high commercial value should be conducted, and reasonable development strategies should be proposed for species whose survival is significantly threatened.

## Data Availability

Please contact the corresponding author for data requests.
